# The First Report of *Elaphostrongylus cervi* Infection in Two Imported Wapitis (*Cervus canadensis*) in Slovenia

**DOI:** 10.3390/vetsci9010019

**Published:** 2022-01-06

**Authors:** Petra Bandelj, Polona Juntes, Gorazd Vengušt, Diana Žele Vengušt

**Affiliations:** Veterinary Faculty, University of Ljubljana, Gerbičeva ulica 60, 1000 Ljubljana, Slovenia; petra.bandelj@vf.uni-lj.si (P.B.); polona.juntes@vf.uni-lj.si (P.J.); gorazd.vengust@vf.uni-lj.si (G.V.)

**Keywords:** *Elaphostrongylus cervi*, tissue worm, wapiti, neurologic, PCR

## Abstract

This study describes two female wapitis (*Cervus canadensis*) with neurological signs associated with an *Elaphostrongylus cervi* (*E. cervi*) infection. The original host of the nematode parasite is the Eurasian red deer (*Cervus elaphus*), although other cervids and small ruminants may also be affected. The two wapitis imported from Canada were kept in an enclosure with the Slovenian red deer herd. After developing debilitating neurological signs, the wapitis were euthanized and examined for possible causes. A histopathological examination of the brain of the first wapiti revealed severe diffuse perivascular meningoencephalitis with chronic vasculitis, and some cross-sections of nematodes were found in the leptomeninges. A necropsy of the second wapiti revealed severe pachymeningitis and leptomeningitis, where several adult nematode parasites were found. *E. cervi* was confirmed by molecular methods. The prevalence of *E. cervi* in the European red deer population is high, but no study has been conducted to assess its prevalence in Slovenia. This was the first confirmation of *E. cervi* in Slovenia and the first infection with this parasite described in Europe in a wapiti. *Elaphostrongylus cervi* should also be considered as a differential diagnosis in Europe for all ruminants grazing on pastures frequented by red deer and showing neurological clinical signs.

## 1. Introduction

*Elaphostrongylus cervi* (Nematoda; Metastrongyloidea; Protostrongylidae, Elaphostrongylinae) (*E. cervi*) is an Old World tissue worm found in the skeletal muscles and central nervous system of cervids, mainly red deer (*Cervus elaphus*) [1,2]. The closely related *E. rangiferi* infests reindeer (*Rangifer tarandus*) [3,4], while *E. alces* is common in moose (*Alces alces*) [5]. The original Eurasian distribution of *E. cervi* has expanded to Canada [6] and New Zealand due to red deer translocation [7], and it has recently been detected in the wild population of Barbary red deer in North Africa [8]. In Europe, *E. cervi* was first reported in Scotland in 1931 [9] and has since been detected in native red deer populations in several countries with a prevalence ranging from 45% to 100% [10,11,12,13]. The prevalence of *E. cervi* in Slovenia is unknown. 

An infection with *E. cervi* usually occurs incidentally in late summer when a deer ingests a terrestrial snail containing the infective third-stage larvae (L3) [14]. During the prepatency period, which lasts three to six months, the L3 larvae migrate from the intestinal wall via the general circulation to the central nervous system [14]. The subsequent larval stage (L4) and adults are located in the subarachnoid spaces from where the adults migrate to the skeletal muscles [1,10,14]. Eggs or newly hatched dorsal spined larvae are carried in the bloodstream to the lungs, where they pass through the alveoli into the bronchial tree. After being coughed up, swallowed, and passed through the gastrointestinal tract, they are excreted into the environment [14]. The first stage larvae (L1) then penetrate through the sole of the snail and develop into the infective L3 stage larvae in about two months [15]. In the final host, clinical signs often occur in winter and are associated with the migration of the parasite [14]. Three forms of the disease have been observed: 1. Neurological with spinal ataxia, asymmetric paraparesis, tetraparesis, blindness, circling, head tilt, and mental confusion, 2. Verminous pneumonia due to larval migration, and 3. Chronic ill health [1,2]. Native cervid species coevolved with the parasite rarely show clinical signs, but non-native deer species and domesticated small ruminants grazing in the same area as red deer can develop severe neurological disease [8,10,16,17,18].

To date, no neurological case in domestic or wild ruminants has been diagnosed in Slovenia due to *E. cervi*. This study describes the first two cases of an *E. cervi* infection detected in Slovenia in two captive wapitis (*Cervus canadensis*) imported from Canada. 

## 2. Case History

On two separate occasions within the same herd of captive-bred wapitis (*Cervus canadensis*), two female wapitis (case one in January 2020 and case two in March 2021) developed debilitating neurological signs that led to the euthanasia (by harvesting) of both animals. The first wapiti was 8 years old and the second was 5 years old at the time of clinical presentation of the disease. The breeding herd (two males, eight females) located in Southeast Slovenia, shares the same enclosure with a herd of 35–40 native red deer (*Cervus elaphus*). The wapiti herd was first imported from a wapiti breeding farm in Canada to a private zoo in the Czech Republic and then translocated to the Slovenian breeder in 2017. The breeder described both females as being in very good condition (approximately 350 kg) and pregnant. Both wapitis developed head tilt, torticollis, circling, ataxia, and loss of balance. Clinical signs developed and worsened so rapidly that within a few days both animals had to be euthanized. Blood samples, heads, and organs were sent to the Veterinary Faculty of the University of Ljubljana for necropsy and the exclusion of possible zoonoses. Appropriate brain samples were collected and tested for chronic wasting disease (CWD), rabies and *Listeria monocytogenes* (*L. monocytogenes*) in the TSE, virology, and microbiology laboratories, respectively. Histopathology of the whole brain and organs with gross lesions was performed after fixation in buffered formalin and paraffin embedding. Slides were stained with hematoxylin and eosin (HE). Blood samples were examined for microelement imbalances and hematological abnormalities. Liver and serum were analyzed for copper, and the intestinal contents were analyzed for gastrointestinal parasites and lungworms. 

## 3. Results

### 3.1. Case 1

#### 3.1.1. Blood and Biochemical Results

Hematology revealed a high white blood cell count (22.2 × 10^9^/L), while biochemistry revealed a slightly low hepatic copper level (3.15 µg/kg or 49.6 µmol/kg). The serum copper levels were low, but within the normal range (198 µg/L or 3.12 µmol/L).

#### 3.1.2. Necropsy and Histopathology

**Figure 1:**
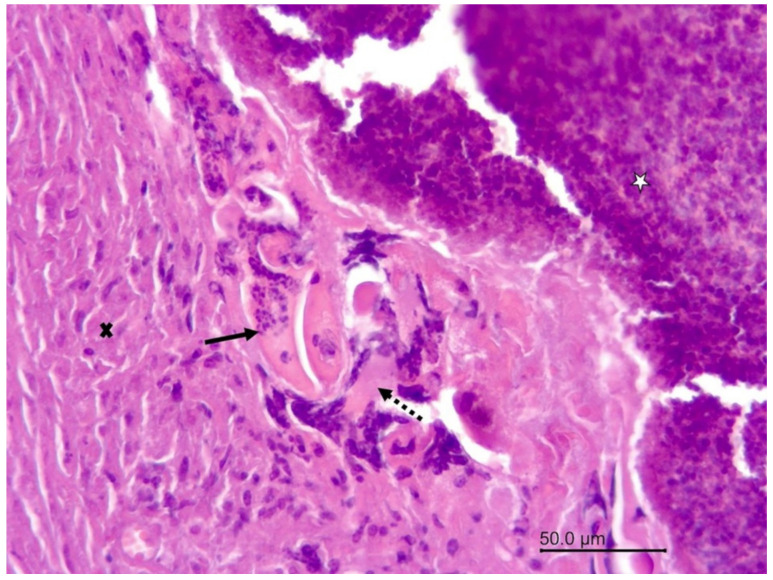
Wapiti, case 1, liver, histopathology, HE, ×200. Structures resembling parasitic larvae (full arrow) between the fibrous capsule (cross) and necrotic inflammatory cells in the center of the granuloma (star), and around these structures are giant cells (dashed arrow) and macrophages.

**Figure 2:**
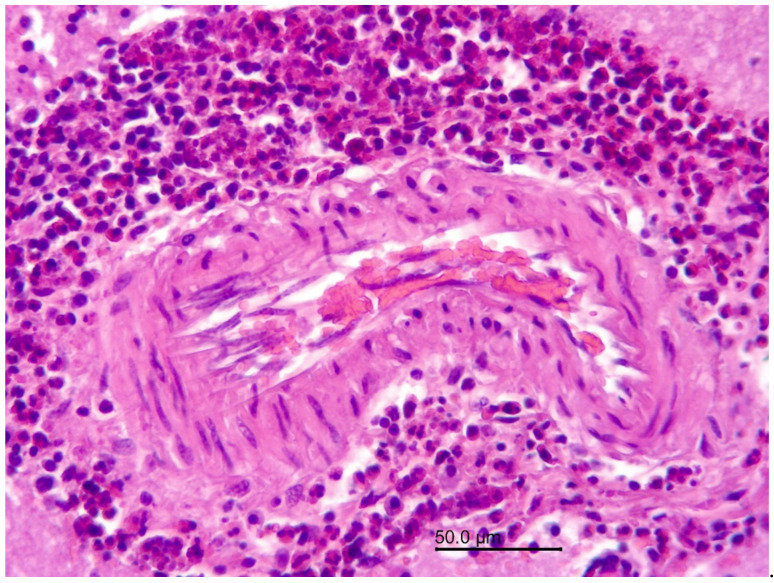
Wapiti, case 1, brain, histopathology, HE, ×200. Blood vessel with a mass of eosinophils in the perivascular space (eosinophilic perivasculitis).

**Figure 3:**
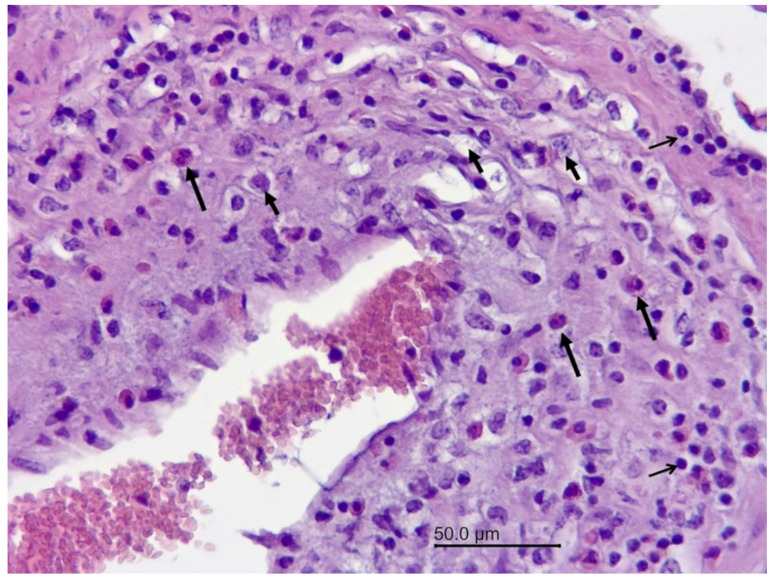
Wapiti, case 1, brain, histopathology, HE, ×200. Blood vessel with numerous inflammatory cells in the wall–eosinophils (long arrows), macrophages (short arrows), and lymphocytes (open arrows) (mixed cell vasculitis).

**Figure 4:**
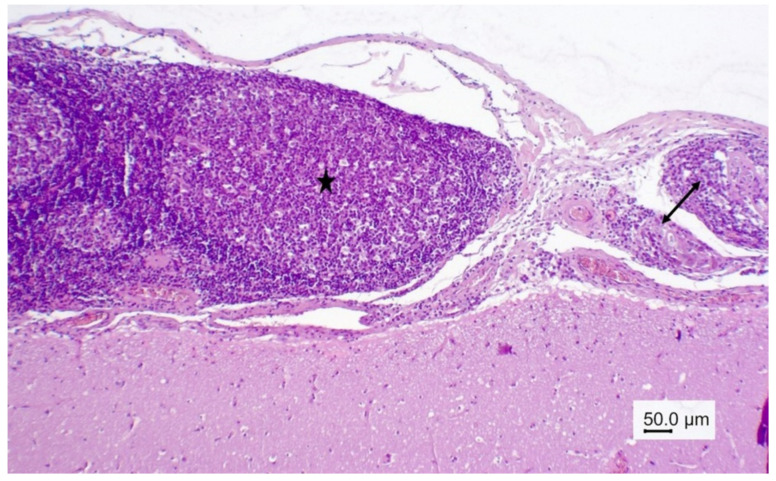
Wapiti, case 1, leptomeninges, histopathology, HE, ×40. Thickened and inflamed leptomeninges. Proliferation of lymphoid follicles with large germinal centers (star) in the leptomeninges on the left, vasculitis and perivasculitis on the right (double arrow).

**Figure 5:**
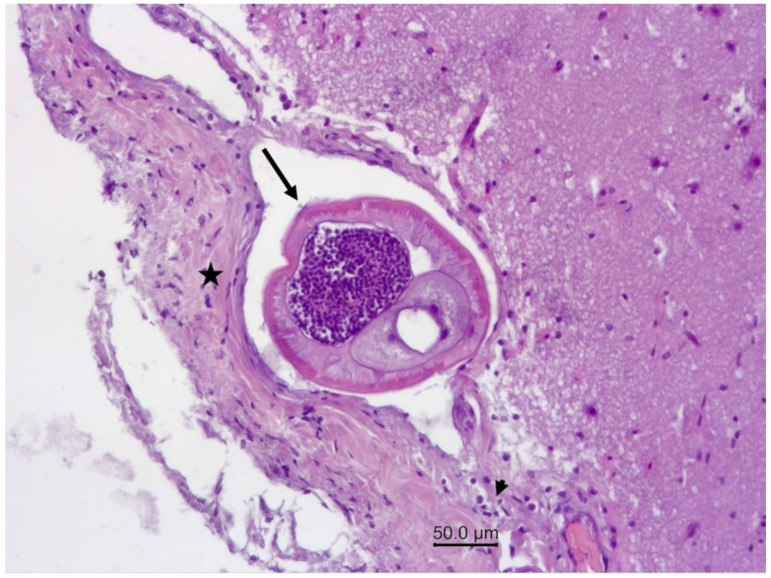
Wapiti, case 1, leptomeninges, histopathology, HE, ×100. Cross-section of a nematode in a vein (arrow). The blood vessel is surrounded by proliferating connective tissue (star) and a small number of inflammatory cells (arrowhead).

The 8-year-old female wapiti tested negative for CWD, rabies, and listeriosis. At necropsy, the internal organs were normal except for the liver, in which numerous firm to hard mineralized white round nodules measuring 0.5 mm–1.5 mm in diameter were found. A histopathological examination confirmed severe chronic miliary granulomatous hepatitis. The granulomas were mostly encapsulated, and many of them were calcified. They contained a mixture of inflammatory cells including neutrophils, eosinophils, lymphocytes, plasma cells, macrophages, and multinucleated giant cells. The granulomas were negative for fungi, acid-fast bacteria, Gram-negative and Gram-positive bacteria, and one contained debris resembling parasitic larvae (Figure 1). An examination of the brain revealed severe inflammatory lesions, most of which were associated with blood vessels. Many blood vessels in the brain and leptomeninges were surrounded by multiple layers of inflammatory cells, often predominated by eosinophils (Figure 2). Other cells included lymphocytes, plasma cells, and macrophages, which frequently infiltrated the blood vessel walls (Figure 3). The vascular lesions were most prominent in the cerebrum and brainstem and less so in the cerebellum and medulla oblongata. The leptomeninges were edematous, and there was a proliferation of lymphoid follicles at several sites (Figure 4). Cross-sections of nematodes were found in the meninges and associated blood vessels at several sites (Figure 5). The diagnosis was parasite-related (verminous) meningoencephalitis and vasculitis.

### 3.2. Case 2

#### 3.2.1. Necropsy and Histopathology

**Figure 6:**
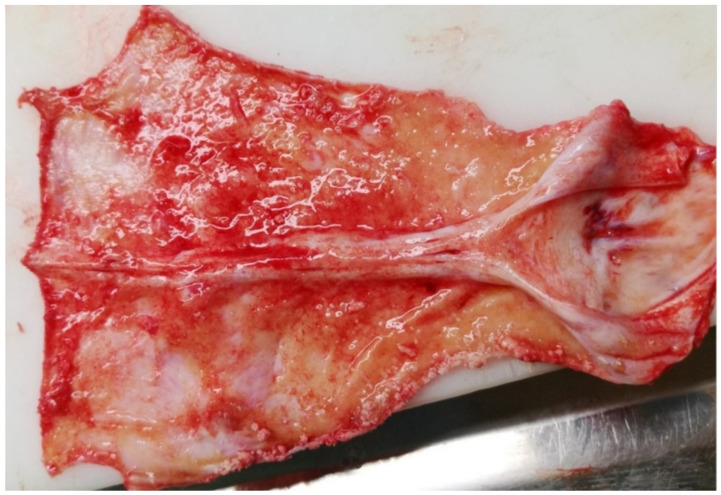
Wapiti, case 2, visceral surface of dura mater, gross pathology. Severe diffuse chronic proliferative and still active acute serofibrinous pachymeningitis.

**Figure 7:**
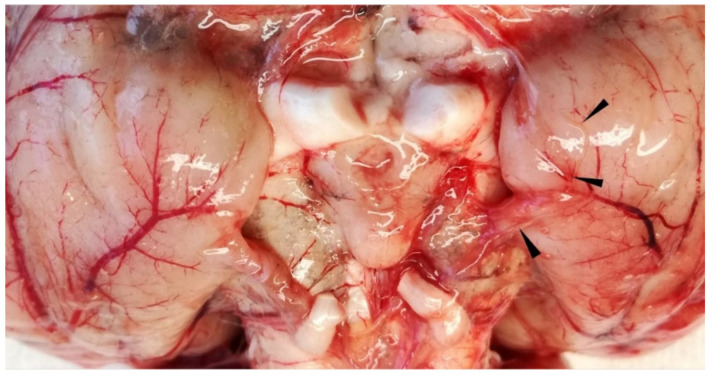
Wapiti, case 2, ventral side of brain, gross pathology. Severe subacute serofibrinous meningitis and thin nematode on surface of piriform lobe partially embedded in blood vessel (arrowheads).

**Figure 8:**
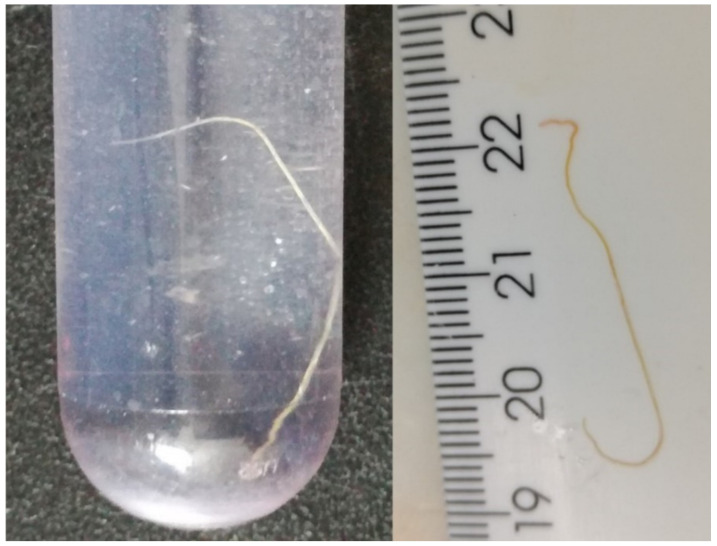
Wapiti, case 2. The nematodes from the meningeal vessels were confirmed by molecular methods as *Elaphostrongylus cervi*.

The 5-year-old female wapiti tested negative for CWD and rabies, but *L. monocytogenes* was isolated from the brain in a culture, although without corresponding morphological lesions in the braincharacteristic of listeriosis. The internal organs, except for the meninges and brain, showed no significant pathological changes. Gross pathology of the brain showed severe chronic pachymeningitis (Figure 6) and leptomeningitis with severe hyperemia and edema (Figure 7). Several nematode parasites were found in the leptomeninges (Figure 7, Figure 8), some of which were collected for parasitological and molecular determination. Histopathology revealed chronic lymphoplasmacytic perivascular inflammation of the dura mater and brain, extending into the medulla oblongata. The leptomeninges were thickened and fibrous and firmly attached to the brain surface. Many blood vessels in the gray and white matter were surrounded by multiple layers of inflammatory cells. However, the vasculitis was less obvious than in case one, and no nematodes were found on histopathological examination, except those found at necropsy. The diagnosis was parasitic pachymeningitis and meningoencephalitis, and the parasitic etiology was supported by the gross pathological findings of nematodes. Other lesions included granulomatous (actinomycotic) lymphadenitis of the retropharyngeal lymph nodes, uneven capsular fibrosis and mild portal fibrosis of the liver, mild smooth muscle hypertrophy of the bronchial and bronchiolar walls, and circumscribed catarrhal bronchiolitis.

#### 3.2.2. Parasitological Examination

The intestinal contents were sent for parasitological examination for gastrointestinal parasites and lungworm larvae. While other coprological tests were negative, Baermann’s method revealed one dorsally spiked first-stage larva (L1) that remained undetermined. 

One of the parasites collected at necropsy and preserved in fixative was female and measured 43 mm (Figure 8), the other was only partially preserved, and the sex could not be determined. To determine the species, the nematodes were then examined by molecular methods.

#### 3.2.3. Molecular Analysis

The DNA from the adult worm was extracted using the SmartHelix First DNAid kit (IFB, Slovenia) following the manufacturer’s instructions [19]. A polymerase chain reaction (PCR) with subsequent sequencing was used as described previously [8,20] for the determination of *E. cervi*. Primers NC1 5′-ACGTCTGGTTCAGGGTTGTT-3′ and NC2 5′-TTAGTTTCTTTTCCTCCGCT-3′ [20] were used for the PCR, amplifying a 597-base-pair (bp) product representing part of the second internal transcribed spacer region (ITS 2) of the ribosomal RNA (rRNA) gene specific for Elaphostrongylinae (*E. cervi*, *E. rangiferi*) (Figure S1). The PCR mix was adjusted to fit a total volume of 25 µL, consisting of 15.65 µL PCR grade H2O, 1 × Taq buffer in 2.5 µL, 2.5 mM MgCl2 in 1.25 µL, 0.25 mM dNTPs in 2.5 µL, 1 µM in 0.25 µL of each primer, 1 U in 0.1 µL Platinum Taq DNA polymerase (Invitrogen, Thermo Fisher, Carlsbad, CA, USA), and 2.5 µL template DNA. The PCR protocol used started with 3 min at 94 °C, continued with 35 × cycles of 94 °C for 1 min, 60 °C for 1 min, 72 °C for 1 min, and finished with 10 min at 72 °C. The PCR yielded one band close to 600 bp. The product was purified with the Wizard PCR Preps DNA Purification System (Promega, Madison, WI, USA) and sent to the Macrogen laboratory (Macrogen Inc, Amsterdam, The Netherlands) for Sanger sequencing. The sequence of the amplified tract was 100% identical to those reported in GenBank for *E. cervi* (accession number AF504032.1; KX066194.1) and confirmed the diagnosis of elaphostrongylosis caused by *E. cervi*.

## 4. Discussion

In our study, we found the presence of *E. cervi* as a most likely cause of neurological disease in two imported wapitis kept in the same enclosure as a herd of native red deer from Slovenia. To the authors’ knowledge, this is the first documented case of an *E. cervi* infection in Slovenia, the first *E. cervi* infection of a wapiti in Europe, and the first molecularly confirmed *E. cervi* infection in a wapiti to date. 

It was reported that clinical signs developed rapidly in the two female wapitis from this study. There were several differential diagnoses that would fit the observed neurological clinical signs, such as listeriosis, copper deficiency, and extrapulmonary lungworms [17,21,22,23]. Testing for transmissible spongiform encephalopathies (for CWD in cervids), rabies, and listeriosis in animals with neurological signs is part of the national surveillance routinely performed, and it was negative for CWD and rabies. *L. monocytogenes* was indeed found in the brain tissue from the 2021 female wapiti. However, no histopathological lesions were found in the brain that could be associated with this bacterium [22], and it was declared an incidental finding. It is also important to note that the 2020 wapiti, in which *L. monocytogenes* was not found, had the same clinical signs as the 2021 wapiti. Copper deficiency was another suggested differential diagnosis, as it can cause enzootic ataxia in captive wild ruminants [23], but the mild deficiency found in the liver of the 2020 wapiti is normal in pregnant female wapitis [24]. The breeder also stated that the animals were in very good body condition and had a healthy coat, making this hypothesis unlikely. 

A necropsy of the 2020 case revealed pathological changes in the liver suggestive of a parasitic etiology but did not immediately indicate that these lesions were related to a neurological disease. When the adult nematode parasites were recovered from the brain samples of the 2021 wapiti, there was sufficient suitable material to apply confirmatory methods. The anatomical location where the worms were found, the histopathological lesions in the brain combined with the clinical signs described in both wapiti led us to two possible diagnoses: *E. cervi* infection and *P. tenuis* infection [17,21].

Epidemiologically, *E. cervi* is the more likely cause of neurological disorders in small ruminants and cervids in Eurasia [10]. Furthermore, the wapiti herd in Slovenia was kept in the same enclosure as approximately 35–40 European red deer, which are thought to be the original host for *E. cervi* as they show almost no clinical signs of infection [2]. Both female wapitis began to show neurological clinical signs during the winter months (January 2020, March 2021), which correlates with the *E. cervi* prepatency period of three to six months [14,25,26]. On the other hand, we could not exclude another very important North American parasite, *P. tenuis*. *P. tenuis* is also known as meningeal worm and is most common in white-tailed deer [3,27,28]. This parasite causes neurological clinical signs in wapiti similar to those of *E. cervi* [21]. Based on the information that the wapiti herd in this case originated from Canada, an infection with *P. tenuis* was a possible diagnosis to consider. 

Morphologically, the adult female of *E. cervi* is between 33 mm–58 mm long [29] and the female of *P. tenuis* is between 40 mm–47 mm long in a wapiti host [30]. The adult female nematode recovered from the 2021 wapiti was 43 mm long and would fit both diagnoses. In one wapiti, the pathological changes caused by nematode parasites were also found in the liver. There are no reports of liver damage during migration of *P. tenuis* [31]. On the other hand, the L3 larval stage of *E. cervi* follows a porto–hepatic pathway from the abomasum and small intestine to the lungs and then through the general circulation to the central nervous system (CNS) [14]. Lymphohistiocytic granulomas and chronic foci of inflammation in liver tissue due to migration of *E. cervi* have already been noted in experimentally infected red deer calves, sheep, and goats [14,32]. Handeland et al. [14] reported in their study that liver damage correlated with the number of L3 larvae ingested. This may explain the liver damage in only one of the wapiti from this study. The wapiti from 2020 may have ingested more L3 larvae of *E. cervi* than the wapiti from 2021, resulting in a mass migration of L3 larvae also through the liver tissue and earlier onset of neurological signs (January versus March). 

The two female wapitis from this study were 8 and 5 years old. Importation from Canada was at least 5 years ago, so the 5-year-old wapiti was most likely born in Europe, and it is very unlikely that the wapiti was infected with *P. tenuis* larvae. Post mortem, we detected a dorsally spined larva in the feces of the wapiti from 2021 using Baermann’s method, but it remained indeterminate. This method has proven unreliable for an antemortem diagnosis of an extrapulmonary lungworm infection because larvae are shed intermittently and in low numbers in aberrant hosts, such as the wapiti [33]. 

Finally, molecular methods confirmed the most likely diagnosis of elaphostrongylosis caused by *E. cervi* in the wapiti from 2021. In the wapiti from 2020, no ancillary test was performed to undoubtedly confirm the same diagnosis. However, due to an almost identical similarity to the wapiti case in 2021, we can safely assume that the most likely diagnosis is also elaphostrongylidae caused by *E. cervi*. The wapiti has been known as a possible host for *E. cervi* since 1976. At that time, two wapitis were diagnosed as infected with *E. cervi* based on the morphological characteristics of the parasite [17]. Interestingly, the present study appears to be the first report of an *E. cervi* infection in wapiti since that time. 

Future *E. cervi* infections of the remaining wapiti herd should be prevented. Deer farmers rely on anthelmintics and changes in management practices to control parasite infections [34,35]. Macrocyclic lactones and benzimidazoles are the most commonly used drugs in small ruminant farms, with a twice/year drenching in spring/summer and fall/winter [35,36]. The wapiti breeder in Slovenia implemented a similar deworming program in his wapiti and red deer herds, using injectable and in-feed macrocyclic lactones (ivermectin) in the fall and an in-feed benzimidazole in the spring. However, one study showed that ivermectin administered via feed only temporarily reduced the shedding of *E. cervi* larvae in red deer, and it was suggested that the adult parasites were still alive and reproducing after treatment [37]. Based on the study by Rodriguez et al. [37], the wapiti herd might have had better results if the red deer herd had been treated with ivermectin in the spring to reduce the number of shedding L1 larvae of *E. cervi,* and if the wapiti herd had been treated with injectable ivermectin in the fall, when the L3 larvae in the wapiti host are in the early migration phase. According to Mitskevich [38] and Panin [39], definitive hosts become infected by accidental ingestion of snails containing L3, mainly in late summer. This may suggest that proper herd management practice, i.e., keeping the wapiti herd in a pasture separate from the red deer herd during late summer, may also help prevent an *E. cervi* infection in wapiti. 

As far as the authors are aware, no case of an *E. cervi* infection has been reported in domestic or wild ruminants in Slovenia. However, some studies have reported the disease in grazing small ruminants in Italy and Switzerland [10,16]. It is suspected that an *E. cervi* infection is misdiagnosed because the clinical signs resemble those of neurological listeriosis. Elaphostrongylosis should be considered as a differential diagnosis when a domestic ruminant or farmed deer exhibits neurological clinical signs and has a history of grazing on pastures frequented by red deer [10]. Based on studies in neighboring countries [10,40,41], the assumption is that the red deer population in Slovenia has a high prevalence of *E. cervi*. However, further studies are needed to assess the prevalence in the resident red deer population and the associated risk for grazing domestic ruminants and farmed mixed herds of deer. 

## 5. Conclusions

This study confirmed the presence of the nematode *E. cervi* in Slovenian wapiti. Both animals, which showed signs of neurological disease, had been imported and shared an enclosure with a herd of native red deer in Slovenia. Elaphostrongylosis was confirmed by necropsy, parasitological and molecular methods. We believe that elaphostrongylosis should be considered as a differential diagnosis for ruminants grazing on pastures frequented by red deer and showing neurological clinical signs.

## Data Availability

The data presented in this study are available on request from the corresponding author.

## Supplementary Materials

The following are available online at https://www.mdpi.com/article/10.3390/vetsci9010019/s1, Figure S1: Capillary electrophoresis results after PCR amplification of a 597-base-pair (bp) product representing a part of the second internal transcribed spacer (ITS 2) region of the ribosomal RNA (rRNA) gene specific for Elaphostrongylinae (*E. cervi*, *E. rangiferi*).
