# Supplementary material for: The First Report of Elaphostrongylus cervi Infection in Two Imported Wapitis (Cervus canadensis) in Slovenia
Source: Vet Sci. 2022 Jan 6;9(1):19. doi: 10.3390/vetsci9010019 (PMC8777883; doi:10.3390/vetsci9010019)
Supplement: Supplementary file 1 [file vetsci-09-00019-s001.zip › vetsci-1502594-supplementary.pdf]

veterinary sciences

MDPI

Supplemental materials:

Capillary electrophoresis results after PCR amplification of a 597-base-pair (bp) product representing a part of the second internal transcribed spacer (ITS 2) region of the ribosomal RNA (rRNA) gene specific for Elaphostrongylinae (*E. cervi, E. rangiferi*).

Petra Bandelj, Polona Juntes, Gorazd Vengušt and Diana Žele Vengušt

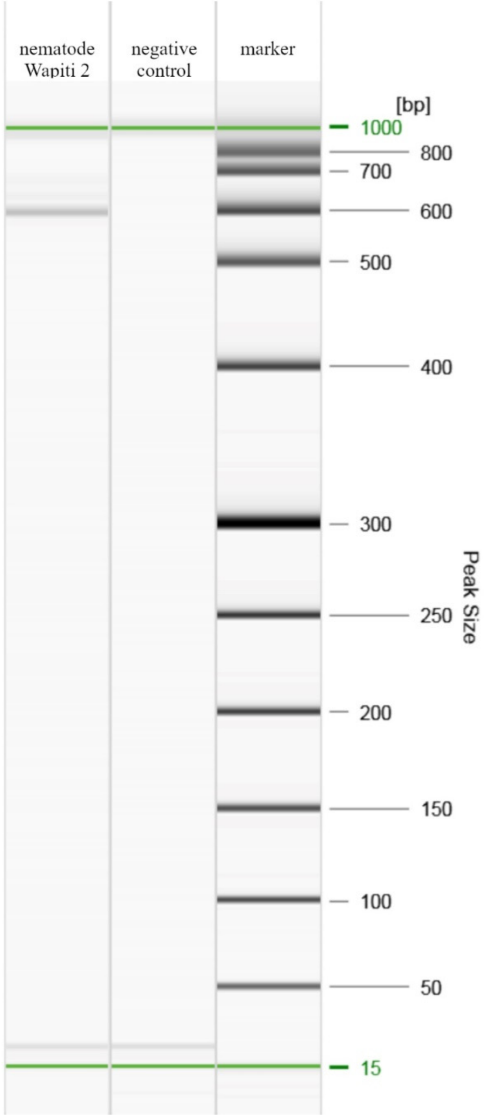

Figure S1. Capillary electrophoresis results after PCR amplification of a 597-base-pair (bp) product representing a part of the second internal transcribed spacer (ITS 2) region of the ribosomal RNA (rRNA) gene specific for Elaphostrongylinae (*E. cervi, E. rangiferi*).
